# Independent Prognosis Prediction of Hepatocellular Carcinoma Using MicroRNA‐Based Single and Combined Biomarkers

**DOI:** 10.1155/cjgh/1491679

**Published:** 2026-06-28

**Authors:** Long-Bin Jeng, Wen-Ling Chan, Chiao-Fang Teng

**Affiliations:** ^1^ Organ Transplantation Center, China Medical University Hospital, Taichung, 404, Taiwan, cmu.edu.tw; ^2^ Department of Surgery, China Medical University Hospital, Taichung, 404, Taiwan, cmu.edu.tw; ^3^ Cell Therapy Center, China Medical University Hospital, Taichung, 404, Taiwan, cmu.edu.tw; ^4^ School of Medicine, China Medical University, Taichung, 404, Taiwan, cmu.edu.tw; ^5^ Bioinformatics and Medical Engineering, College of Medical and Health Science, Asia University, Taichung, 413, Taiwan, asia.edu.tw; ^6^ Graduate Institute of Biomedical Sciences, China Medical University, Taichung, 404, Taiwan, cmu.edu.cn; ^7^ Master Program for Cancer Biology and Drug Discovery, China Medical University, Taichung, 404, Taiwan, cmu.edu.tw; ^8^ Cancer Biology and Precision Therapeutics Center, China Medical University, Taichung, 404, Taiwan, cmu.edu.tw

**Keywords:** combined, hepatocellular carcinoma, independent prognostic biomarker, microRNA, single

## Abstract

Although there are various treatment modalities available for hepatocellular carcinoma (HCC), HCC is still among the most common causes of cancer‐related death globally. Identifying independent biomarkers of poor prognosis in HCC patients remains a critical goal to allow timely intervention and ameliorate patient survival. MicroRNAs (miRNAs), the most widely investigated small noncoding RNAs to date, play a crucial role in regulating the initiation and progression of HCC. The expression of numerous miRNAs is altered in tumor tissues and blood specimens of HCC patients, underscoring their potential as promising prognostic biomarkers. This review offers an exhaustive overview of the current literature examining tissue‐ and circulation‐derived miRNAs as single or combined independent prognostic biomarkers in HCC patients.

## 1. Introduction

Hepatocellular carcinoma (HCC) is the most prevalent form of primary liver cancer, representing approximately 90% of all hepatic tumors. Globally, HCC ranks sixth among the most frequent human cancers, causing nearly 900,000 new cases annually, or 4.3% of all cancer cases [1–3]. Numerous established therapeutic options are available, including surgery, localized therapies, and systemic drugs; however, the survival outcomes related to these treatments are suboptimal [4–6]. The current cure rate is about 25%, with an overall 5‐year survival rate of less than 30% [7, 8]. HCC is the third most common cause of mortality from cancer worldwide, causing as many as 800,000 deaths per year (7.8% of all cancer deaths) [2, 9]. Given that HCC progresses rapidly and is frequently diagnosed at an advanced stage, the mortality rate of HCC closely aligns with its incidence rate, with a worldwide mortality‐to‐incidence ratio up to 0.86 [2, 3]. Hence, the identification and development of independent prognostic biomarkers are essential for earlier disease detection and faster therapeutic intervention, thereby enhancing patient survival.

In clinical practice, determining the prognosis of HCC patients relies on a multifaceted approach that integrates tumor burden, hepatic functional reserve, and patient performance status. Traditional staging systems, such as the Barcelona Clinic Liver Cancer (BCLC) staging, the Hong Kong Liver Cancer classification, and the Cancer of the Liver Italian Program score, remain the cornerstones for predicting survival outcomes and guiding therapeutic strategies [10–12]. Additionally, serum biomarkers play a vital role; elevated baseline levels of albumin‐bilirubin grade, alpha‐fetoprotein (AFP), and des‐gamma‐carboxy prothrombin are heavily associated with poor prognosis and early recurrence [13–15]. In recent years, the prognostic landscape has expanded to include advanced molecular and digital tools. Liquid biopsies, which detect circulating tumor cells and cell‐free DNA mutations, offer minimally invasive, real‐time monitoring of tumor evolution [16, 17]. Furthermore, radiomics and artificial intelligence algorithms are increasingly applied to preoperative computed tomography and magnetic resonance imaging scans to decode complex tumor phenotypes, predict microvascular invasion (MVI), and calculate patient‐specific survival trajectories [18, 19].

MicroRNAs (miRNAs or miRs), the most extensively studied small noncoding RNAs, act as critical post‐transcriptional regulators of gene expression [20, 21]. By facilitating the binding of RNA‐induced silencing complexes to complementary sites in the 3′‐untranslated region (UTR) of target messenger RNAs (mRNAs), miRNAs induce mRNA breakdown or inhibit protein synthesis [20, 21]. In addition, miRNAs play key roles in the initial development and malignant progression of various cancers by regulating the expression of oncogenic and tumor suppressor genes [22, 23]. During liver tumorigenesis, miRNAs act as key regulators of essential cellular mechanisms, such as cell cycle control, cell proliferation, apoptosis evasion, epithelial‐to‐mesenchymal transition, migration, invasion, and angiogenesis, by post‐transcriptionally suppressing key tumor suppressors or oncogenes [24, 25]. As HCC progresses, specific miRNA expression profiles correlate directly with aggressive clinicopathological features, including MVI, poor tumor differentiation, and early recurrence [26, 27]. Dysregulated miRNA expression occurs at different stages of HCC development, from early chronic hepatitis and steatosis to cirrhosis and final tumorigenesis [28, 29].

Moreover, miRNAs extracted from tissues or blood in free or exosome‐enclosed forms hold considerable potential as cancer biomarkers owing to their exceptional sample stability, rapid extraction, high tissue specificity, and precise tracking of disease progression [30, 31]. By reflecting the expression levels and pathological status of their respective source tissues and being more readily detectable, tissue mRNAs provide distinct advantages over circulating miRNAs [32]. However, circulating miRNA detection requires less invasive and safer sample collection procedures, supporting their clinical applicability. Moreover, exosome‐enclosed miRNAs exhibit higher stability than that of free‐circulating miRNAs under various storage conditions, further demonstrating their suitability as biomarkers [33].

Although traditional prognostic tools, such as serum AFP levels, transaminase monitoring, and liver echography, are widely utilized in clinical practice, they exhibit critical limitations. Serum AFP often demonstrates suboptimal sensitivity and specificity, frequently failing to detect early stage tumors or accurately predict post‐therapeutic recurrence [34]. Similarly, transaminase fluctuations reflect general hepatic inflammation rather than tumor kinetics, while the efficacy of liver echography is highly operator‐dependent and limited in identifying microlesions [35, 36]. To overcome these prognostic blind spots, tissue‐ and circulation‐derived miRNAs have emerged as powerful, next‐generation prognostic biomarkers. Compared with protein‐based assays, nucleic acid detection methods are generally more cost‐effective and efficient [30, 31]. Accordingly, numerous miRNAs with altered expression in tumor tissues or blood have been investigated as valuable, next‐generation biomarkers for independent prediction of prognosis in HCC patients [37, 38].

The primary aim of this review is to evaluate the clinical utility of miRNA‐based biomarkers as prognostic tools for HCC. Establishing a clear prognosis is vital for aligning therapeutic interventions with realistic patient outcomes, thereby reducing the risks of both overtreatment and therapeutic nihilism. By systematically summarizing the existing literature that validates the independent prognostic value of multiple single and combined miRNA biomarkers derived from tissues and circulation in HCC patients, this review seeks to provide a comprehensive framework for integrating prognostic insights into clinical practice.

## 2. Tissue‐Derived miRNA Biomarkers as Independent Prognostic Indicators in HCC

Multiple studies have employed multivariate Cox regression analysis to determine the prognostic value of individual miRNAs based on their pretreatment expression in tumor tissues of HCC patients (Table 1). Among the 60 tissue‐derived single‐miRNA biomarkers identified, 30 served as positive predictors, indicating a direct association between their expression levels and patient prognosis. For example, Chen et al. analyzed data from RNA sequencing (RNA‐seq) of 344 retrospectively recruited patients with HCC (166 patients aged ≤ 60 years and 178 patients aged > 60 years) and found that high expression of *let-7c-5p* was correlated with longer overall survival (OS) (hazard ratio [HR]: 0.761; 95% confidence interval [CI]: 0.624−0.929; *p* = 0.007) [39]. Similarly, Chang et al. performed quantitative reverse‐transcription polymerase chain reaction (qRT‐PCR) to analyze postoperative samples from 50 HCC patients (mean age ± standard deviation [SD], 65.3 ± 8.9 years for 25 patients with high *miR-26a-5p* expression and 62.7 ± 9.5 years for 25 patients with low *miR-26a-5p* expression), revealing a positive association between upregulated *miR-26a-5p* expression and prolonged OS (HR: 0.532; 95% CI: 0.221–1.312; *p* = 0.001) [40]. Liang et al. confirmed through qRT‐PCR that high *miR-27b* expression predicts better OS (HR: 0.335; 95% CI: 0.230–0.548; *p* = 0.007) in 68 HCC patients (28 patients aged < 50 years and 40 patients aged ≥ 50 years) following curative surgical resection [41].

**TABLE 1:** Independent prognostic significance of tissue‐derived single‐miRNA biomarkers in patients with HCC.

Biomarker	Sampling source	Detection method	Study design	Patients analyzed	Prognostic significance	Year	References
*Positive predictors*							
*let-7c-5p*	Tumor tissue	RNA‐seq	Retrospective	344 patients with HCC	High *let-7c-5p* level predicted longer OS	2022	Chen et al. [39]

*miR-26a-5p*	Tumor tissue	qRT‐PCR	Retrospective	50 patients with HCC treated with curative surgical resection	High *miR-26a-5p* level predicted longer OS	2017	Chang et al. [40]

*miR-27b*	Tumor tissue	qRT‐PCR	Retrospective	68 patients with HCC treated with curative surgical resection	High *miR-27b* level predicted longer OS	2019	Liang et al. [41]

*miR-122*	Tumor tissue	qRT‐PCR	Retrospective	289 patients with HCC treated with curative surgical resection	Low *miR-122* level predicted shorter RFS	2019	Ha et al. [42]

*miR-124*	Tumor tissue	qRT‐PCR	Retrospective	131 patients with HCC treated with curative surgical resection	High *miR-124* level predicted longer DFS	2012	Zheng et al. [43]

*miR-128-3p*	Tumor tissue	qRT‐PCR	Retrospective	72 patients with HCC treated with curative surgical resection	High *miR-128-3p* level predicted longer OS	2015	Huang et al. [44]

*miR-129-5p*	Tumor tissue	qRT‐PCR	Retrospective	106 patients with HCC treated with curative surgical resection	Low *miR-129-5p* level predicted shorter OS and DFS	2016	Liu et al. [45]

*miR-130a*	Tumor tissue	qRT‐PCR	Retrospective	102 patients with HCC treated with curative surgical resection	Low *miR-130a* level predicted shorter OS	2014	Li et al. [46]

*miR-139-3p*	Tumor tissue	RNA‐seq	Retrospective	344 patients with HCC	High *miR-139-3p* level predicted longer OS	2021	Zhang et al. [47]

*miR-139-5p*	Tumor tissue	RNA‐seq	Retrospective	375 patients with HCC	High *miR-139-5p* level predicted longer OS	2019	Wang et al. [48]

*miR-145*	Tumor tissue	qRT‐PCR	Retrospective	139 patients with HCC treated with curative surgical resection	Low *miR-145* level predicted shorter OS	2018	Li et al. [49]

*miR-192-5p*	Tumor tissue	qRT‐PCR	Retrospective	101 patients with HCC treated with curative surgical resection	Low *miR-192-5p* level predicted shorter OS	2016	Lian et al. [50]

*miR-197-3p*	Tumor tissue	qRT‐PCR	Retrospective	197 patients with HCC treated with curative surgical resection	Low *miR-197-3p* level predicted shorter RFS	2019	Ni et al. [51]

*miR-203*	Tumor tissue	qRT‐PCR	Retrospective	66 patients with HCC treated with liver transplantation	High *miR-203* level predicted longer OS and RFS	2012	Chen et al. [52]

*miR-218*	Tumor tissue	qRT‐PCR	Retrospective	60 patients with HCC treated with curative surgical resection	Low *miR-218* level predicted shorter OS and DFS	2014	Tu et al. [53]

*miR-223-3p*	Tumor tissue	qRT‐PCR	Retrospective	70 patients with HBV‐related HCC treated with curative surgical resection	Low *miR-223-3p* level predicted shorter OS	2020	Pratedrat et al. [54]

*miR-302a-3p*	Tumor tissue	qRT‐PCR	Retrospective	111 patients with HCC treated with curative surgical resection	High *miR-302a-3p* level predicted longer OS	2018	Ye et al. [55]

*miR-342-3p*	Tumor tissue	qRT‐PCR	Retrospective	164 patients with HCC treated with curative surgical resection	Low *miR-342-3p* level predicted shorter OS	2017	Gao et al. [56]

*miR-370*	Tumor tissue	qRT‐PCR	Retrospective	83 patients with HCC treated with curative surgical resection	Low *miR-370* level predicted shorter OS	2017	Pan et al. [57]

*miR-424-5p*	Tumor tissue	qRT‐PCR	Retrospective	90 patients with HCC treated with curative surgical resection	Low *miR-424-5p* level predicted shorter OS	2019	Du et al. [58]

*miR-451*	Tumor tissue	qRT‐PCR	Retrospective	88 patients with HCC treated with curative surgical resection	Low *miR-451* level predicted shorter OS	2015	Huang et al. [59]

*miR-501*	Tumor tissue	qRT‐PCR	Retrospective	53 patients with HCC treated with curative surgical resection	Low *miR-501* level predicted shorter OS	2019	Yu et al. [60]

*miR-506-3p*	Tumor tissue	RNA‐seq	Retrospective	376 patients with HCC	High *miR-506-3p* level predicted longer OS	2021	Li et al. [61]

*miR-517b-3p*	Tumor tissue	qRT‐PCR	Retrospective	124 patients with HCC treated with curative surgical resection	Low *miR-517b-3p* level predicted shorter OS	2022	Ke et al. [62]

*miR-526b*	Tumor tissue	qRT‐PCR	Retrospective	80 patients with HCC treated with curative surgical resection	High *miR-526b* level predicted longer OS	2017	Liu et al. [63]

*miR-655-3p*	Tumor tissue	qRT‐PCR	Retrospective	188 patients with HCC treated with curative surgical resection	Low *miR-655-3p* level predicted shorter OS	2017	Zhao et al. [64]

*miR-744*	Tumor tissue	qRT‐PCR	Retrospective	96 patients with HCC treated with liver transplantation	Low *miR-744* level predicted shorter OS and RFS	2015	Tan et al. [65]

*miR-3607*	Tumor tissue	qRT‐PCR	Retrospective	122 patients with HCC treated with curative surgical resection	Low *miR-3607* level predicted shorter OS	2020	Dou et al. [66]

*miR-4772-3p*	Tumor tissue	RNA‐seq	Retrospective	376 patients with HCC	High *miR-4772-3p* level predicted longer OS	2021	Li et al. [61]

*miR-375*	Tumor tissue	qRT‐PCR	Retrospective	38 patients with HCC treated with curative surgical resection	Low *miR-375* level predicted shorter DFS	2016	Zhou et al. [67]
	Tumor tissue	qRT‐PCR	Retrospective	70 patients with HCC treated with curative surgical resection	High *miR-375* level predicted longer OS	2017	Xie et al. [68]

*Negative Predictors*							
*miR-9*	Tumor tissue	qRT‐PCR	Retrospective	200 patients with HCC treated with curative surgical resection	High *miR-9* level predicted shorter OS	2014	Cai and Cai [69]

*miR-17-5p*	Tumor tissue	qRT‐PCR	Retrospective	120 patients with HCC treated with curative surgical resection	High *miR-17-5p* level predicted shorter OS and DFS	2012	Chen et al. [70]

*miR-18a*	Tumor tissue	qRT‐PCR	Retrospective	123 patients with HCC treated with curative surgical resection	High *miR-18a* level predicted shorter OS	2018	Wang et al. [71]

*miR-23b-3p*	Tumor tissue	qRT‐PCR	Retrospective	125 patients with HCC treated with curative surgical resection	High *miR-23b-3p* level predicted shorter OS	2021	Hayashi et al. [72]

*miR-25*	Tumor tissue	qRT‐PCR	Retrospective	131 patients with HCC treated with curative surgical resection	High *miR-25* level predicted shorter OS	2014	Su et al. [73]

*miR-92a*	Tumor tissue	qRT‐PCR	Retrospective	106 patients with HCC treated with curative surgical resection	High *miR-92a* level predicted shorter OS and RFS	2015	Yang et al. [74]

*miR-130b*	Tumor tissue	qRT‐PCR	Retrospective	97 patients with HCC treated with curative surgical resection	High *miR-130b* level predicted shorter OS and DFS	2014	Wang et al. [75]

*miR-137*	Tumor tissue	qRT‐PCR	Retrospective	99 patients with HCC treated with curative surgical resection	High *miR-137* level predicted shorter OS and CSS	2017	Sakabe et al. [76]

*miR-141-3p*	Tumor tissue	RNA‐seq	Retrospective	376 patients with HCC	High *miR-141-3p* level predicted shorter OS	2021	Li et al. [61]

*miR-219-5p*	Tumor tissue	qRT‐PCR	Retrospective	191 patients with HCC treated with curative surgical resection	High *miR-219-5p* level predicted shorter OS and RFS	2018	Yang et al. [77]

*miR-326*	Tumor tissue	RNA‐seq	Retrospective	344 patients with HCC	High *miR-326* level predicted shorter OS	2022	Chen et al. [39]

*miR-346*	Tumor tissue	RNA‐seq	Retrospective	376 patients with HCC	High *miR-346* level predicted shorter OS	2021	Li et al. [61]

*miR-425-5p*	Tumor tissue	qRT‐PCR	Retrospective	110 patients with HCC treated with curative surgical resection	High *miR-425-5p* level predicted shorter OS and DFS	2017	Fang et al. [78]

*miR-509-3-5p*	Tumor tissue	RNA‐seq	Retrospective	376 patients with HCC	High *miR-509-3-5p* level predicted shorter OS	2021	Li et al. [61]

*miR-522*	Tumor tissue	qRT‐PCR	Retrospective	161 patients with HCC treated with curative surgical resection	High *miR-522* level predicted shorter OS	2016	Shi et al. [79]

*miR-548f-3p*	Tumor tissue	RNA‐seq	Retrospective	376 patients with HCC	High *miR-548f-3p* level predicted shorter OS	2021	Li et al. [61]

*miR-654-3p*	Tumor tissue	qRT‐PCR	Retrospective	117 patients with HCC treated with curative surgical resection	High *miR-654-3p* level predicted shorter OS	2020	Yang et al. [80]

*miR-760*	Tumor tissue	RNA‐seq	Retrospective	344 patients with HCC	High *miR-760* level predicted shorter OS	2021	Zhang et al. [47]

*miR-937*	Tumor tissue	qRT‐PCR	Retrospective	125 patients with HCC treated with curative surgical resection	High *miR-937* level predicted shorter OS	2022	Chen and Zhang [81]

*miR-1180-3p*	Tumor tissue	RNA‐seq	Retrospective	88 patients with HCC	High *miR-1180-3p* level predicted shorter OS	2020	Zhou et al. [82]

*miR-3180*	Tumor tissue	RNA‐seq	Retrospective	32 patients with HCC treated with curative surgical resection	Low *miR-3180* level predicted longer OS	2023	Sun et al. [83]

*miR-3677-5p*	Tumor tissue	qRT‐PCR	Retrospective	80 patients with HCC treated with curative surgical resection	High *miR-3677-5p* level predicted shorter OS and RFS	2021	Mao et al. [84]

*miR-3682*	Tumor tissue	RNA‐seq	Retrospective	231 patients with HCC	High *miR-3682* level predicted shorter OS	2022	Zhao and Liu [85]

*miR-5010-3p*	Tumor tissue	RNA‐seq	Retrospective	372 patients with HCC	High *miR-5010-3p* level predicted shorter OS	2023	Cao et al. [86]

*miR-7702*	Tumor tissue	RNA‐seq	Retrospective	376 patients with HCC	High *miR-7702* level predicted shorter OS	2021	Li et al. [61]

*miR-7-5p*	Tumor tissue	RNA‐seq	Retrospective	344 patients with HCC	High *miR-7-5p* level predicted shorter OS	2022	Chen et al. [39]
	Tumor tissue	RNA‐seq	Retrospective	344 patients with HCC	High *miR-7-5p* level predicted shorter OS	2021	Zhang et al. [47]

*miR-21*	Tumor tissue	qRT‐PCR	Retrospective	119 patients with HCC treated with curative surgical resection	High *miR-21* level predicted shorter OS and DFS	2014	Wang et al. [87]
	Tumor tissue	qRT‐PCR	Retrospective	112 patients with HCC treated with curative surgical resection	High *miR-21* level predicted shorter OS	2015	Huang et al. [88]

*miR-106b-5p*	Tumor tissue	qRT‐PCR	Retrospective	104 patients with HCC treated with curative surgical resection	High *miR-106b-5p* level predicted shorter OS	2014	Li et al. [89]
	Tumor tissue	qRT‐PCR	Retrospective	108 patients with HCC treated with curative surgical resection	High *miR-106b-5p* level predicted shorter OS and RFS	2019	Gu et al. [90]

*miR-221*	Tumor tissue	qRT‐PCR	Retrospective	135 patients with HCC treated with curative surgical resection	High *miR-221* level predicted shorter OS and DFS	2017	Chen et al. [91]
	Tumor tissue	qRT‐PCR	Retrospective	70 patients with HCC treated with curative surgical resection	High *miR-221* level predicted shorter OS	2017	Xie et al. [68]

*miR-1266-5p*	Tumor tissue	qRT‐PCR	Retrospective	119 patients with HCC treated with curative surgical resection	High *miR-1266-5p* level predicted shorter OS	2021	Huang et al. [92]
	Tumor tissue	qRT‐PCR	Retrospective	132 patients with HCC treated with curative surgical resection	High *miR-1266-5p* level predicted shorter OS	2021	Su et al. [93]

*Note:* miRNA, microRNA; HCC, hepatocellular carcinoma.

Abbreviations: CSS, cancer‐specific survival; DFS, disease‐free survival; HBV, hepatitis B virus; OS, overall survival; qRT‐PCR, quantitative reverse‐transcription polymerase chain reaction; RFS, recurrence‐free survival; RNA‐seq, RNA sequencing.

Furthermore, Ha et al. demonstrated that low *miR-122* expression, measured by qRT‐PCR in 289 retrospectively recruited HCC patients after curative surgical resection (167 patients aged ≤ 55 years and 122 patients aged > 55 years; median age [range], 53 (17−76) years), predicts shorter recurrence‐free survival (RFS) (HR: 1.75; 95% CI: 1.11−2.75; *p* = 0.016) [42]. Moreover, high *miR-124* expression was correlated with longer disease‐free survival (DFS) (HR: 0.4; 95% CI: 0.2−0.8; *p* = 0.009) among 131 HCC patients receiving curative surgical resection (63 patients aged ≤ 50.3 years and 68 patients aged > 50.3 years) [43]. Similarly, elevated *miR-128-3p* expression levels were linked to better OS (HR: 0.323; 95% CI: 0.121–0.864; *p* = 0.024) in 72 HCC patients following surgery (47 patients aged < 50 years and 25 patients aged ≥ 50 years) [44]. Furthermore, low *miR-129-5p* expression predicted poorer OS (HR: 2.152; 95% CI: 1.237−3.685; *p* = 0.004) and DFS (HR: 3.527; 95% CI: 1.634−7.753; *p* = 0.001) in 106 HCC patients postsurgery (51 patients aged < 65 years and 55 patients aged ≥ 65 years) [45].

Moreover, reduced *miR-130a* expression predicted shorter OS (HR: 2.217; 95% CI: 1.103–4.458; *p* = 0.025) in 102 HCC patients undergoing curative surgical resection (48 patients aged < 50 years and 54 patients aged ≥ 50 years) [46]. High *miR-139-3p* expression was also correlated with longer OS (HR: 0.772; 95% CI: 0.642–0.928; *p* = 0.006) in 344 retrospectively recruited HCC patients [47]. In addition, upregulated *miR-139-5p* expression was correlated with extended OS (HR: 0.69; 95% CI: 0.53−0.9; *p* = 0.007) in 375 HCC patients [48]. Downregulated *miR-145* expression predicted shorter OS (HR: 2.126; 95% CI: 1.529−10.273; *p* = 0.033) in 139 HCC patients postresection (64 patients aged ≤ 60 years and 75 patients aged > 60 years) [49].

Additional research includes the identification of high *miR-192-5p* expression as a predictor of longer OS (HR: 3.739; 95% CI: 1.127−12.407; *p* = 0.031) in 101 HCC patients [50] and low *miR-197-3p* expression as the predictor of shorter RFS (HR: 1.448; 95% CI: 1.039–2.018; *p* = 0.029) in 197 HCC patients following resection (104 patients aged < 55 years and 93 patients aged ≥ 55 years) [51]. Furthermore, high *miR-203* expression predicted improved OS (HR: 0.332; 95% CI: 0.139–0.793; *p* = 0.013) and RFS (HR: 0.202; 95% CI: 0.064–0.638; *p* = 0.006) in 66 HCC patients receiving liver transplantation (30 patients aged ≤ 55 years and 36 patients aged > 55 years) [52].

Low *miR-218* expression predicted shorter OS (HR: 3.475; 95% CI: 1.515−7.972; *p* = 0.003) and DFS (HR: 2.547; 95% CI: 1.240−5.232; *p* = 0.011) in 60 HCC patients following curative surgical resection [53]. However, high *miR-223-3p* levels were linked to longer OS (HR: 6.61; 95% CI: 2.36−18.55; *p* < 0.001) in 70 hepatitis B virus (HBV)–related HCC patients following curative surgical resection (mean age ± SD, 52.0 ± 8.3 years) [54]. Ye et al. established that heightened *miR-302a-3p* expression predicted longer OS (HR: 0.480; 95% CI: 0.249–0.894; *p* = 0.039) in 111 HCC patients (62 patients aged ≤ 50 years and 49 patients aged > 50 years) [55].

Lower expression of various miRNAs has also been correlated with reduced OS in HCC patients following surgical resection, including *miR-342-3p* in 164 patients (95 patients aged < 60 years and 69 patients aged ≥ 60 years) (HR: 2.231; 95% CI: 1.234–4.893; *p* = 0.002) [56], *miR-370* in 83 patients (34 patients aged ≤ 50 years and 49 patients aged > 50 years) (HR: 2.365; 95% CI: 1.015–9.324; *p* = 0.016) [57], *miR-424-5p* in 90 patients (43 patients aged < 50 years and 47 patients aged ≥ 50 years) (HR: 1.023; 95% CI: 1.220–2.039; *p* = 0.010) [58], and *miR-451* in 88 patients (39 patients aged ≤ 55 years and 49 patients aged > 55 years) (HR: 2.09; 95% CI: 1.20−3.63; *p* = 0.009) [59]. Similarly, lower *miR-501* expression predicted poorer OS (HR: 1.08; 95% CI: 0.78−8.65; *p* = 0.032) in 53 retrospectively recruited patients with HCC treated with curative surgical resection (31 patients aged < 60 years and 22 patients aged ≥ 60 years) [60].

In 376 retrospectively enrolled HCC patients, the upregulation of miR‐506‐3p was correlated with prolonged OS (HR: 0.797; 95% CI: 0.626−0.926; *p* = 0.043) [61]. Meanwhile, the downregulation of *miR-517b-3p* predicted shorter OS (HR: 3.137; 95% CI: 1.570–6.269; *p* = 0.001) in 124 HCC patients after curative surgical resection (49 patients aged ≤ 50 years and 75 patients aged > 50 years) [62]. Liu et al. concluded that high *miR-526b* predicted better OS (HR: 0.255; 95% CI: 0.082–0.076; *p* = 0.021) in 80 HCC patients (32 patients aged ≤ 50 years and 48 patients aged > 50 years) [63], whereas Zhao et al. noted that low *miR-655-3p* expression predicted shorter OS (HR: 1.533; 95% CI: 0.988–3.891; *p* = 0.002) in 188 HCC patients (69 patients aged < 60 years and 119 patients aged ≥ 60 years) [64]. Similarly, low *miR-744* expression predicted poorer OS (HR: 4.177; 95% CI: 1.670–10.452; *p* = 0.002) and RFS (HR: 4.196; 95% CI: 1.736–10.138; *p* = 0.001) in 96 HCC patients who underwent liver transplantation (mean age ± SD, 54.0 ± 8.3 years for 48 patients with high *miR-744* expression; 51.5 ± 8.9 years for 48 patients with low *miR-744* expression) [65]. Low *miR-3607* expression was also associated with shorter OS (HR: 2.005; 95% CI: 1.089–3.882; *p* = 0.035) in 122 HCC patients (44 patients aged ≤ 50 years and 78 patients aged > 50 years) [66]. In contrast, high *miR-4772-3p* expression was correlated with longer OS (HR: 0.915; 95% CI: 0.874−0.955; *p* = 0.004) in 376 HCC patients [61].

Two studies have validated the independent prognostic performance of certain miRNA biomarkers. Zhou et al. showed that low expression of miR‐375 was associated with shorter DFS (HR: 3.273; 95% CI: 1.107–9.679; *p* = 0.032) in 38 patients with HCC postoperatively (24 patients aged < 60 years and 14 patients aged ≥ 60 years) [67]. Conversely, high *miR-375* expression was correlated with longer OS (HR: 0.153; 95% CI: 0.035–0.837; *p* = 0.014) in 70 patients with HCC who underwent curative resection (29 patients aged < 50 years and 41 patients aged ≥ 50 years) [68].

The remaining 30 tissue‐derived single‐miRNA biomarkers serve as negative predictors, exhibiting inverse associations with patient prognosis. For example, Cai et al. unraveled that high *miR-9* expression predicted reduced OS (HR: 4.28; 95% CI: 2.77−7.23; *p* < 0.001) in 200 retrospectively recruited HCC patients after curative surgical resection (78 patients aged < 50 years and 122 patients aged ≥ 50 years) [69]. Similarly, higher *miR-17-5p* expression was identified as a predictor of reduced OS (HR: 4.96; 95% CI: 1.78−13.82; *p* = 0.002) and DFS (HR: 1.79; 95% CI: 1.14−2.98; *p* = 0.042) in 120 HCC patients (96 patients aged ≤ 65 years and 24 patients aged > 65 years) [70]. Numerous other miRNAs exhibited similar trends in HCC patients receiving curative surgical resection, with higher expression level predicting poorer OS: *miR-18a* in 123 patients (33 patients aged ≤ 50 years and 90 patients aged > 50 years) (HR: 2.017; 95% CI: 1.137–3.578; *p* = 0.017) [71], *miR-23b-3p* in 125 patients (52 patients aged < 65 years and 73 patients aged ≥ 65 years) (HR: 2.43; 95% CI: 1.32−4.47; *p* = 0.004) [72], and *miR-25* in 131 patients (53 patients aged ≤ 50 years and 78 patients aged > 50 years) (HR: 2.179; 95% CI: 1.876–4.335; *p* = 0.001) [73].

Upregulated *miR-92a* expression was also correlated with shorter OS (HR: 2.283; 95% CI: 1.104–4.717; *p* = 0.026) and RFS (HR: 3.706; 95% CI: 1.079–5.155; *p* = 0.042) in 106 HCC patients (42 patients aged ≤ 50 years and 64 patients aged > 50 years) [74]. Moreover, high expression of *miR-130b* was a predictor of shorter OS (HR: 2.523; 95% CI: 1.024–7.901; *p* = 0.011) and DFS (HR: 4.003; 95% CI: 1.578–7.889; *p* = 0.005) in 97 HCC patients (46 patients aged < 50 years and 51 patients aged ≥ 50 years) [75]. Sakabe et al. demonstrated high *miR-137* expression as an indicator of shorter OS (HR: 2.050; 95% CI: 1.074–3.914; *p* = 0.030) and cancer‐specific survival (CSS) (HR: 2.206; 95% CI: 1.005–4.844; *p* = 0.049) in 99 patients with HCC after surgical resection [76].

Furthermore, high expression of miR‐141‐3p was associated with a shorter OS (HR: 1.128; 95% CI: 1.096−1.135; *p* = 0.009) in 376 HCC patients [61]. Similarly, high *miR-219-5p* expression was correlated with reduced OS (HR: 1.689; 95% CI: 1.433–3.903; *p* = 0.036) and RFS (HR: 1.663; 95% CI: 1.072–2.577; *p* = 0.023) in 191 HCC patients (129 patients aged < 50 years and 62 patients aged ≥ 50 years) [77]. In a large study comprising 344 HCC patients (166 patients aged ≤ 60 years and 178 patients aged > 60 years), high *miR-326* expression was identified as a predictor of shortened OS (HR: 1.317; 95% CI: 1.035−1.676; *p* = 0.025) [39]. Similarly, high *miR-346* expression was correlated with shorter OS (HR: 1.140; 95% CI: 1.105–1.175; *p* = 0.024) in 376 HCC patients [61]. Fang et al. verified that high *miR-425-5p* expression predicted shorter OS (HR: 2.857; 95% CI: 1.555–5.247; *p* = 0.001) and DFS (HR: 2.414; 95% CI: 1.394–4.180; *p* = 0.002) in 110 HCC patients following curative surgical resection (85 patients aged ≤ 60 years and 25 patients aged > 60 years) [78].

Similar inverse relationships have been reported between shorter OS and elevated expression of *miR-509-3-5p* in HCC 376 patients (HR: 1.079; 95% CI: 1.015–1.159; *p* = 0.036) [61], *miR-522* in 161 HCC patients (66 patients aged < 50 years and 95 patients aged ≥ 50 years) (HR: 1.37; 95% CI: 1.09−5.14; *p* = 0.002) [79], *miR-548f-3p* in 376 HCC patients (HR: 1.107; 95% CI: 1.102−1.113; *p* = 0.001) [61], *miR-654-3p* in 117 HCC patients (57 patients aged ≤ 55 years and 60 patients aged > 55 years) (HR: 1.891; 95% CI: 1.057−3.381; *p* = 0.032) [80], *miR-760* in 344 HCC patients (HR: 1.293; 95% CI: 1.050–1.592; *p* = 0.015) [47], *miR-937* in 125 HCC patients (61 patients aged ≤ 55 years and 64 patients aged > 55 years) (HR: 3.237; 95% CI: 1.385–7.567; *p* = 0.007) [81], and *miR-1180-3p* in 88 HCC patients (47 patients aged ≤ 54 years and 41 patients aged > 54 years) (HR: 1.25; 95% CI: 1.07−1.45; *p* = 0.004) [82].

In contrast, low *miR-3180* expression predicted longer OS (HR: 0.08; 95% CI: 0.01−0.59; *p* = 0.013) in 32 HCC patients following curative surgical resection (12 patients aged < 50 years and 20 patients aged ≥ 50 years) [83]. Meanwhile, high *miR-3677-5p* expression predicted poorer OS (HR: 1.632; 95% CI: 1.287−2.015; *p* = 0.019) and RFS (HR: 1.781; 95% CI: 1.210−2.337; *p* = 0.033) in 80 HCC patients (38 patients aged < 60 years and 42 patients aged ≥ 60 years) [84]. High *miR-3682* expression was demonstrated as a predictor of shorter OS (HR: 2.213; 95% CI: 1.361–3.598; *p* = 0.001) in 231 HCC patients (121 patients aged < 60 years and 110 patients aged ≥ 60 years) [85]. Similar trends were observed for *miR-5010-3p* in 372 HCC patients (OS, HR: 1.957; 95% CI: 1.192−3.211; *p* = 0.008) [86] and *miR-7702* in 376 HCC patients (OS, HR: 1.327; 95% CI: 1.215–1.339; *p* < 0.001) [61].

In addition, two different studies demonstrated that certain miRNA biomarkers work as independent prognostic indicators. Chen et al. confirmed that high *miR-7-5p* expression detected by RNA‐seq was associated with poorer OS (HR: 1.296; 95% CI: 1.045–1.607; *p* = 0.018) in 344 retrospectively recruited patients with HCC (166 patients aged ≤ 60 years and 178 patients aged > 60 years) [39]. Using RNA‐seq–based detection of miR‐7‐5p, Zhang et al. ascertained that upregulated *miR-7-5p* expression was correlated with reduced OS (HR: 1.359; 95% CI: 1.112–1.660; *p* = 0.003) in 344 retrospectively recruited HCC patients [47].

Wang et al. demonstrated high *miR-21* expression as a predictor of shorter OS (HR: 3.189; 95% CI: 1.911−10.012; *p* = 0.03) and DFS (HR: 5.897; 95% CI: 3.009−13.763; *p* < 0.001) in 119 HCC patients (54 patients aged < 50 years and 65 patients aged ≥ 50 years) [87]. Similarly, the upregulation of *miR-21* in 112 HCC patients (49 patients aged < 50 years and 63 patients aged ≥ 50 years) (HR: 2.275; 95% CI: 1.394–7.924; *p* = 0.005) [88] and *miR-106b-5p* in 104 HCC patients (49 patients aged < 50 years and 55 patients aged ≥ 50 years) (HR: 2.002; 95% CI: 1.130–6.977; *p* = 0.027) [89] was associated with shorter OS. Meanwhile, high *miR-106b-5p* expression predicted shortened OS (HR: 7.268; 95% CI: 1.122–21.260; *p* = 0.01) and RFS (HR: 5.101; 95% CI: 1.328–12.968; *p* = 0.02) in 108 HCC patients (48 patients aged < 50 years and 60 patients aged ≥ 50 years) [90]. Chen et al. found that high *miR-221* expression was correlated with poorer OS (HR: 2.969; 95% CI: 1.629−5.408; *p* < 0.001) and DFS (HR: 2.846; 95% CI: 1.564−5.181; *p* = 0.001) in 135 HCC patients (mean age ± SD, 51.1 ± 9.4 years for 62 patients with high *miR-221* expression; 53.3 ± 9.1 years for 73 patients with low *miR-221* expression) [91]. Similar relationships were reported between upregulated miRNA expression and shorter OS by Xie et al. for *miR-221* in 70 HCC patients (29 patients aged < 50 years and 41 patients aged ≥ 50 years) (HR: 1.743; 95% CI: 1.004–3.772; *p* = 0.012) [68], Huang et al. for *miR-1266-5p* in 119 HCC patients (58 patients aged < 60 years and 61 patients aged ≥ 60 years) (HR: 2.048; 95% CI: 1.151–3.644; *p* = 0.015) [92], and Su et al. for *miR-1266-5p* in 132 HCC patients (52 patients aged ≤ 50 years and 80 patients aged > 50 years) (HR: 2.359; 95% CI: 1.462−3.807; *p* < 0.001) [93].

## 3. Prognostic Value of Circulating Single‐miRNA Biomarkers in HCC

Multiple studies have applied multivariate Cox regression analysis to assess the prognostic value of specific miRNAs whose pretreatment blood levels are altered in HCC patients (Table 2). Among the 21 circulating single‐miRNA biomarkers, 10 serve as positive predictors, exhibiting a direct positive association between their expression and patient prognosis. For example, Koberle et al. identified a higher serum level of *miR-1-3p* as a predictor of longer OS (HR: 0.451; 95% CI: 0.238−0.856; *p* = 0.015) in 195 prospectively recruited HCC patients (mean age ± SD, 62.6 ± 10.4 years) [94]. In contrast, Cho et al. reported that a low plasma *miR-26a-5p* level was correlated with poorer DFS (HR: 1.72; 95% CI: 1.04−2.83; *p* = 0.035) and a low serum *miR-29a-3p* level predicted a shorter DFS (HR: 1.75; 95% CI: 1.04−2.94; *p* = 0.035) in 120 retrospectively recruited HBV‐related HCC patients undergoing curative surgical resection or radiofrequency ablation (RFA) (mean age ± SD, 54.0 ± 9.9 years) [95]. In a retrospective cohort of 60 HCC patients treated with curative surgical resection (24 patients aged ≤ 55 years and 36 patients aged > 55 years), Chen et al. reported an association between low serum exosomal *miR-34a* expression and reduced OS (HR: 1.918; 95% CI: 1.009−3.646; *p* = 0.047) [96]. Wang et al. determined that downregulated serum *miR-148a* expression was a predictor of shorter OS (HR: 2.261; 95% CI: 1.084−4.718; *p* = 0.030) in 76 retrospectively recruited HCC patients receiving curative surgical resection (38 patients aged < 51 years and 38 patients aged ≥ 51 years) [97]. Hao et al. verified the correlation between low serum exosomal *miR-320a* expression and shorter OS (HR: 2.974; 95% CI: 1.562–4.628; *p* = 0.008) in 104 retrospectively recruited HCC patients receiving curative surgical resection (48 patients aged < 60 years and 56 patients aged ≥ 60 years) [98]. Similarly, low *miR-320d* expression in serum exosomes predicted shorter OS (HR: 2.85; 95% CI: 1.18−5.69; *p* = 0.021) in 110 retrospectively recruited HCC patients (47 patients aged ≤ 60 years and 63 patients aged > 60 years) [99]. In contrast, high serum *miR-497* expression was associated with longer OS (HR: 0.014; 95% CI: 0.0−0.787; *p* = 0.038) in 60 HCC patients treated with curative surgical resection (mean age ± SD, 50.1 ± 7.7 years) [100]. In 110 HBV‐related HCC patients (mean age ± SD, 57.7 ± 6.0 years), low serum *miR-768-3p* expression predicted shorter OS (HR: 3.057; 95% CI: 1.136−8.225; *p* = 0.027) [101].

**TABLE 2:** Independent prognostic significance of circulation‐derived single‐miRNA biomarkers in patients with HCC.

Biomarker	Sampling source	Detection method	Study design	Patients analyzed	Prognostic significance	Year	References
*Positive predictor*							
*miR-1-3p*	Serum	qRT‐PCR	Prospective	195 patients with HCC	High *miR-1-3p* level predicted longer OS	2013	Koberle et al. [94]

*miR-26a-5p*	Plasma	qRT‐PCR	Retrospective	120 patients with HBV‐related HCC treated with curative surgical resection or RFA	Low *miR-26a-5p* level predicted shorter DFS	2017	Cho et al. [95]

*miR-29a-3p*	Plasma	qRT‐PCR	Retrospective	120 patients with HBV‐related HCC treated with curative surgical resection or RFA	Low *miR-29a-3p* level predicted shorter DFS	2017	Cho et al. [95]

*miR-34a*	Serum exosome	qRT‐PCR	Retrospective	60 patients with HCC treated with curative surgical resection	Low *miR-34a* level predicted shorter OS	2022	Chen et al. [96]

*miR-148a*	Serum	qRT‐PCR	Retrospective	76 patients with HCC treated with curative surgical resection	Low *miR-148a* level predicted shorter OS	2016	Wang et al. [97]

*miR-320a*	Serum exosome	qRT‐PCR	Retrospective	104 patients with HCC treated with curative surgical resection	Low *miR-320a* level predicted shorter OS	2020	Hao et al. [98]

*miR-320d*	Serum exosome	qRT‐PCR	Retrospective	110 patients with HCC	Low *miR-320d* level predicted shorter OS	2020	Li et al. [99]

*miR-497*	Serum	qRT‐PCR	Retrospective	60 patients with HCC treated with curative surgical resection	High *miR-497* level predicted longer OS	2021	Ali et al. [100]

*miR-768-3p*	Serum	qRT‐PCR	Retrospective	110 patients with HBV‐related HCC	Low *miR-768-3p* level predicted shorter OS	2020	Cao and Wang [101]

*miR-638*	Serum exosome	qRT‐PCR	Retrospective	126 patients with HCC treated with curative surgical resection	Low *miR-638* level predicted shorter OS	2018	Shi et al. [102]
	Serum exosome	qRT‐PCR	Retrospective	54 patients with HCC treated with curative surgical resection	Low *miR-638* level predicted shorter DFS	2021	Yokota et al. [103]

*Negative Predictor*							
*miR-19-3p*	Plasma exosome	qRT‐PCR	Retrospective	70 patients with non‐HBV–/non‐HCV–related HCC	High *miR-19-3p* level predicted shorter OS	2023	Boonkaew et al. [104]

*miR-21*	Serum exosome	qRT‐PCR	Prospective	79 patients with HCC	High *miR-21* level predicted shorter OS	2019	Lee et al. [105]

*miR-24-3p*	Serum	qRT‐PCR	Retrospective	72 patients with HBV‐related HCC treated with curative surgical resection	High *miR-24-3p* level predicted shorter OS and DFS	2014	Meng et al. [106]

*miR-182*	Serum	qRT‐PCR	Retrospective	103 patients with HCC treated with curative surgical resection	High *miR-182* level predicted shorter OS	2015	Chen et al. [107]

*miR-192-5p*	Serum	qRT‐PCR	Retrospective	74 patients with HBV‐related HCC treated with curative surgical resection and/or RFA or TACE	High *miR-192-5p* level predicted shorter OS and PFS	2016	Zhu et al. [108]

*miR-210*	Serum	qRT‐PCR	Retrospective	113 patients with HCC treated with TACE	High *miR-210* level predicted shorter OS	2014	Zhan et al. [109]

*miR-218*	Serum	qRT‐PCR	Retrospective	156 patients with HCC treated with curative surgical resection	High *miR-218* level predicted shorter OS	2016	Yang et al. [110]

*miR-221*	Serum	qRT‐PCR	Retrospective	46 patients with HCC	High *miR-221* level predicted shorter OS	2011	Li et al. [111]

*miR-331-3p*	Serum	qRT‐PCR	Retrospective	103 patients with HCC treated with curative surgical resection	High *miR-331-3p* level predicted shorter OS	2015	Chen et al. [107]

*miR-487b*	Serum	qRT‐PCR	Retrospective	87 patients with HBV‐related HCC treated with curative surgical resection	High *miR-487b* level predicted shorter OS	2020	Cao et al. [112]
	Serum	qRT‐PCR	Retrospective	116 patients with HBV‐related HCC treated with curative surgical resection	High *miR-487b* level predicted shorter OS	2021	Li et al. [113]

*miR-1246*	Plasma	qRT‐PCR	Retrospective	62 patients with HCC treated with liver transplantation	High *miR-1246* level predicted shorter OS and DFS	2016	Ng et al. [114]
	Serum	Microarray	Retrospective	121 patients with HCC treated with curative surgical resection	High *miR-1246* level predicted shorter OS	2019	Chuma et al. [115]

*Note:* miRNA, microRNA; HCC, hepatocellular carcinoma; RFA, radiofrequency ablation; TACE, transarterial chemoembolization.

Abbreviations: qRT‐PCR, quantitative reverse‐transcription polymerase chain reaction; OS, overall survival; DFS, disease‐free survival; HBV, hepatitis B virus; HCV, hepatitis C virus; PFS, progression‐free survival.

Two separate studies have been performed to validate the independent prognostic performance of *miR-638*. Shi et al. confirmed an association between low serum exosomal *miR-638* expression and shortened OS (HR: 3.52; 95% CI: 1.37−6.02; *p* = 0.009) in 126 HCC patients following curative surgical resection (68 patients aged < 65 years and 58 patients aged ≥ 65 years) [102]. Meanwhile, Yokota et al. identified a correlation between low serum exosomal *miR-638* expression and poorer DFS (HR: 3.2128; 95% CI: 1.2972–7.9567; *p* = 0.0117) in 54 HCC patients receiving curative surgical resection (median age [range], 73 (56−84) years for 27 patients with high *miR-638* expression; 70 (56−81) years for 27 patients with low *miR-638* expression) [103].

In contrast, 11 single circulation‐derived miRNA biomarkers have been reported to act as negative predictors, indicating a negative relationship between their expression levels and patient prognosis. In a cohort of 70 retrospectively recruited patients with HCC not related to HBV or hepatitis C virus (HCV) infection (mean age ± SD, 68.8 ± 11.4 years), high expression of *miR-19-3p* in plasma exosomes predicted poorer OS (HR: 2.71; 95% CI: 1.19−6.19; *p* = 0.018) [104]. A similar relationship was observed for high serum exosomal *miR-21* expression in and shorter OS (HR: 2.869; 95% CI: 1.249−6.593; *p* = 0.013) in 79 prospectively recruited HCC patients (median age [range], 59 (52−67) years) [105]. Similarly, Meng et al. verified that high expression of *miR-24-3p* in serum was correlated with shorter OS (HR: 2.141; 95% CI: 1.158–3.960; *p* = 0.015) and DFS (HR: 2.055; 95% CI: 1.114–3.792; *p* = 0.021) in 72 retrospectively recruited HBV‐related HCC patients receiving curative surgical resection (47 patients aged ≤ 60 years and 25 patients aged > 60 years) [106]. High serum miR‐182 expression was also correlated with reduced OS (HR: 2.321; 95% CI: 1.136–4.741; *p* = 0.021) in 103 HCC patients after curative surgical resection (median age [range], 52 (39−80) years) [107].

Zhu et al. reported that increased serum *miR-192-5p* expression predicted poorer OS (HR: 4.0; 95% CI: 1.2−13.9; *p* = 0.027) and progression‐free survival (PFS, HR: 2.2; 95% CI: 1.1−4.2; *p* = 0.023) in 74 HBV‐related HCC patients receiving curative surgical resection and/or RFA or transarterial chemoembolization (TACE) [108]. Zhan et al. found an association between high serum *miR-210* expression and shorter OS (HR: 2.082; 95% CI: 1.281–3.383; *p* = 0.003) in 113 HCC patients following TACE (51 patients aged ≤ 55 years and 62 patients aged > 55 years) [109]. In 156 HCC patients receiving curative surgical resection (97 patients aged ≤ 60 years and 59 patients aged > 60 years), Yang et al. verified that high serum *miR-218* expression was correlated with shorter OS (HR: 3.049; 95% CI: 2.028−4.585; *p* < 0.01) [110]. Similarly, high serum *miR-221* expression in 46 HCC patients (18 patients aged < 50 years and 28 patients aged ≥ 50 years) (HR: 1.903; 95% CI: 1.235−2.981; *p* = 0.018) [111] and *miR-331-3p* expression in 103 HCC patients (median age [range], 52 (39−80) years) (HR: 2.010; 95% CI: 1.043–3.871; *p* = 0.037) [107] were the predictors of shorter OS.

Certain miRNA biomarkers have been validated as independent prognostic indicators in two separate studies. For example, high serum *miR-487b* expression was shown to be correlated with poorer OS by Cao et al. (HR: 2.846; 95% CI: 1.139−7.114; *p* = 0.025; 87 patients (mean age ± SD, 60.0 ± 8.1 years)) [112] and Li et al. (HR: 2.115; 95% CI: 1.083–4.132; *p* = 0.028; 116 patients (mean age ± SD, 56.1 ± 5.5 years)) [113] in patients with HBV‐related HCC treated with curative surgical resection. Ng et al. confirmed that high expression of *miR-1246* in plasma predicted shorter OS (HR: 10.24; 95% CI: 1.39−75.67; *p* = 0.023) and DFS (HR: 10.12; 95% CI: 1.45−70.47; *p* = 0.020) in 62 patients with HCC treated with liver transplantation (median age [range], 55 [30−67] years) [114]. Similarly, Chuma et al. used microarray‐based assays to show that high serum *miR-1246* expression predicted shorter OS (HR: 3.421; 95% CI: 1.327–8.819; *p* = 0.0109) in 121 HCC patients following curative surgical resection (57 patients aged < 67 years and 64 patients aged ≥ 67 years) [115].

## 4. Prognostic Value of miRNA Combination Biomarkers in HCC

Several studies have used multivariate Cox regression analysis to investigate the independent prognostic value of combining different miRNAs with altered pretreatment expression levels in blood samples or tumor tissues of HCC patients (Table 3).

**TABLE 3:** Independent prognostic significance of miRNA‐based combination biomarkers in patients with HCC.

Biomarkers	Sampling source	Detection method	Study design	Patients analyzed	Prognostic significance	Year	References
*Negative predictors*							
Combination of *miR-29a-3p, miR-192-5p*, and BCLC stage	Serum	qRT‐PCR	Retrospective	74 patients with HBV‐related HCC treated with curative surgical resection and/or RFA or TACE	High risk score predicted shorter OS and PFS	2016	Zhu et al. [108]
Combination of *miR-9, miR-187, miR-490, miR-551a, miR-665, miR-1258,* and *miR-3144*	Tumor tissue	RNA‐seq	Retrospective	318 patients with HCC	High risk score predicted shorter OS	2018	Fu et al. [116]
Combination of *miR-26a‐1‐3p, miR-105‐5p, miR-132‐5p, miR-149‐5p, miR-188‐5p,* and *miR-212‐5p*	Tumor tissue	RNA‐seq	Retrospective	433 patients with HCC	High risk score predicted shorter OS	2020	Fang et al. [117]
Combination of *miR-141-3p, miR-346, miR-506-3p, miR-509-3-5p, miR-548f-3p, miR-4772-3p*, and *miR-7702*	Tumor tissue	RNA‐seq	Retrospective	376 patients with HCC	High risk score predicted shorter OS	2021	Li et al. [61]
Combination of *miR-7-5p, miR-139-3p,* and *miR-760*	Tumor tissue	RNA‐seq	Retrospective	344 patients with HCC	High risk score predicted shorter OS	2021	Zhang et al. [47]
Combination of *let-7c-5p, miR-7-5p, miR-30d-5p, miR-326, miR-760,* and *miR-5003-3p*	Tumor tissue	RNA‐seq	Retrospective	344 patients with HCC	High risk score predicted shorter OS	2022	Chen et al. [39]
Combination of *miR-15a-5p, miR-30a-5p, miR-126-3p, miR-148a-3p,* and *miR-199a-5p*	Tumor tissue	RNA‐seq	Retrospective	373 patients with HCC	High risk score predicted shorter OS	2022	Chen et al. [118]

*Note:* miRNA, microRNA; HCC, hepatocellular carcinoma; RFA, radiofrequency ablation; TACE, transarterial chemoembolization.

Abbreviations: BCLC, Barcelona Clinic Liver Cancer; HBV, hepatitis B virus; OS, overall survival; PFS, progression‐free survival; qRT‐PCR, quantitative reverse‐transcription polymerase chain reaction; RNA‐seq, RNA sequencing.

All seven of the identified multi‐miRNA biomarkers showed a negative association between risk scores and patient prognosis; one was derived from the circulation, whereas the remaining six were derived from tissue samples. Zhu et al. used qRT‐PCR to assess two serum miRNAs (*miR-29a-3p* and *miR-192-5p*) combined with the BCLC stage as a prognostic signature, revealing that high risk scores were correlated with shorter OS (HR: 5.2; 95% CI: 2.6−10.9; *p* < 0.001) and PFS (HR: 3.1; 95% CI: 2.0−4.7; *p* < 0.001) in 74 retrospectively recruited patients with HBV‐related HCC treated with curative surgical resection and/or RFA or TACE [108]. Fu et al. measured the expression of seven miRNAs (*miR-9, miR-187, miR-490, miR-551a, miR-665, miR-1258*, and *miR-3144*) in tumor tissues and found that high risk scores predicted shorter OS (HR: 1.034; 95% CI: 1.009−1.06; *p* = 0.007) in 318 retrospectively recruited HCC patients (177 patients aged < 65 years and 141 patients aged ≥ 65 years) [116]. Similarly, Fang et al. analyzed six tumor‐tissue miRNAs (*miR-26a‐1‐3p, miR-105‐5p, miR-132‐5p, miR-149‐5p, miR-188‐5p,* and *miR-212‐5p*) in 433 HCC patients (171 patients aged ≤ 62 years and 162 patients aged > 62 years), confirming that elevated risk scores were correlated with reduced OS (HR: 3.03; 95% CI: 2.07−4.43; *p* < 0.001) [117].

Li et al. assessed seven miRNAs (*miR-141-3p, miR-346, miR-506-3p, miR-509-3-5p, miR-548f-3p, miR-4772-3p,* and *miR-7702*) in tumor tissues as prognostic signatures for shorter OS (HR: 2.769; 95% CI: 1.903–4.029; *p* < 0.001) in 376 retrospectively recruited HCC patients [61]. Similarly, Zhang et al. evaluated the prognostic performance of three tumor‐tissue miRNAs (*miR-7-5p, miR-139-3p,* and *miR-760*), confirming the association between a high signature score and poorer OS (HR: 4.342; 95% CI: 2.326−8.107; *p* < 0.0001) in 344 patients with HCC [47]. Similarly, high signature scores for six tumor‐tissue miRNAs (*let-7c-5p, miR-7-5p, miR-30d-5p, miR-326, miR-760,* and *miR-5003-3p*) predicted shortened OS (HR: 1.367; 95% CI: 1.126–1.659; *p* = 0.002) in 344 HCC patients (166 patients aged ≤ 60 years and 178 patients aged > 60 years) [39]. Five tumor‐tissue miRNAs (*miR-15a-5p, miR-30a-5p, miR-126-3p, miR-148a-3p,* and *miR-199a-5p*) (HR: 1.496; 95% CI: 1.281–1.747; *p* < 0.001) showed similar associations with OS in 373 HCC patients [118].

## 5. Conclusions

**FIGURE 1:**
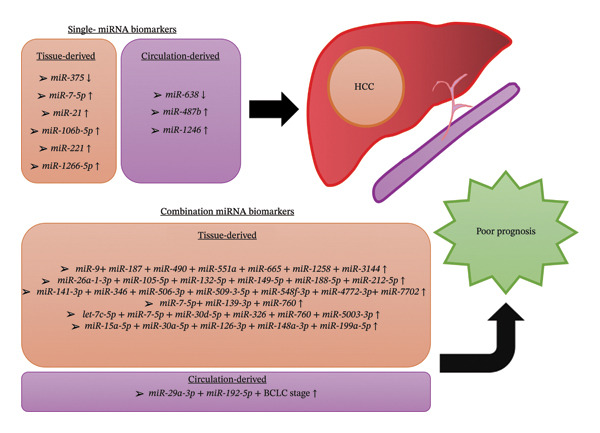
Schematic summary of tissue‐ and circulation‐derived single and combined miRNA biomarkers with independent prognostic value in HCC patients. High or low expression levels of single‐miRNA biomarkers and high risk scores of combined miRNA biomarkers in tumor tissues or serum/plasma/exosome samples independently predict poor prognosis in patients with HCC. The single‐miRNA biomarkers validated by two different lines of studies are shown as representatives. Abbreviations: HCC, hepatocellular carcinoma; miRNA, microRNA.

The studies synthesized in this review support the independent prognostic value of many tissue‐ and circulation‐derived single and combined miRNA biomarkers in HCC patients (Figure 1). Current evidence supports the promising role of these miRNA biomarkers in pinpointing patients with HCC at high risk of poor outcomes, allowing timely and optimized management strategies to improve survival rates. In particular, although circulation‐derived *miR-1-3p* and *miR-21* have been validated in prospective studies, most data for the other miRNAs originate from single‐center retrospective cohort studies using disparate methodologies, which may have led to the overestimation of biomarker performance.

Furthermore, various etiological factors, including HBV, HCV, metabolic‐associated steatotic liver disease (MASLD), and alcohol‐related liver disease (ALD), contribute to HCC pathogenesis and can substantially influence miRNA expression profiles [119–122]. Several studies have specifically analyzed HBV‐related HCC or included HBV factors in multivariate analyses; however, no research has examined the influence of MASLD or ALD on the prognostic utility of these biomarkers. To establish robust and unbiased evidence supporting the clinical application of miRNA biomarkers, prospective multicenter cohort studies with comprehensive stratification of patient characteristics (such as etiology, pathological status, and treatment‐related mortality) are warranted.

Certain miRNAs, including *miR-21*, *miR-26a-5p*, *miR-192-5p*, *miR-218*, and *miR-221*, have been validated as tissue and circulation‐derived biomarkers, demonstrating consistency in predicting prognosis and thus allowing the detection of expression profiles across different sample sources in clinical settings. As miRNAs are increasingly recognized as potential therapeutic targets for cancer [24, 123], additional research is required to evaluate their potential role in identifying patients with HCC suitable for miRNA‐targeted therapies. Given the variability in therapeutic regimens among patient populations, it is essential to select miRNA biomarkers that offer better prognostic accuracy for individualized outcome prediction.

In addition, the precision and reproducibility of current miRNA‐based prognostic biomarkers must be assessed in terms of cohort size, clinicopathological diversity, methodological differences, and research design. Small cohort sizes (approximately 30–70 patients) in some studies can undermine statistical power, and wide CIs for HRs could exaggerate the results. Except for tissue‐derived *miR-21, miR-106b-5p, miR-221, miR-375,* and *miR-1266-5p* and circulation‐derived *miR-487b, miR-638,* and *miR-1246*, which have been evaluated in two separate studies, the prognostic value of most miRNA biomarkers is based on a single study. Although combined miRNA biomarkers have also generally been evaluated in single studies, many combinations have incorporated specific prognostic miRNA markers validated in multiple studies, improving robustness and generalizability. Differences in patient cohorts, with respect to clinicopathological features and treatments, further complicate the interpretation of biomarker efficacy, and the heterogeneity in detection methodologies introduces additional technical variability.

Implementing standardized protocols and workflows for miRNA detection, including sample acquisition and storage, extraction and measurement, and data analysis, is essential to improve reliability and support clinical translation. Recent research has predominantly explored the relationship between miRNA expression and patient prognosis, underscoring the necessity for mechanistic and biological research to clarify the therapeutic value of miRNAs in patients with HCC. Furthermore, miRNA expression levels naturally fluctuate in response to stimuli within the tumor microenvironment and under varying physiological and pathological conditions. As current miRNA biomarkers for the prognosis of HCC are detected primarily before treatment, future direction should include the development of dynamic miRNA panels tailored to individual patient profiles possibly via artificial intelligence‐enhanced bioinformatic approaches.

## Author Contributions

Conceptualization and visualization: Long‐Bin Jeng, Wen‐Ling Chan, and Chiao‐Fang Teng; writing–original draft, funding acquisition, and writing–review and editing: Chiao‐Fang Teng.

## Funding

This work was supported by grants from the China Medical University, Taichung, Taiwan (Grant Nos. CMU112‐ASIA‐06 and CMU114‐ASIA‐03).

## Conflicts of Interest

The authors declare no conflict of interest.

## Data Availability

Data sharing is not applicable to this article as no datasets were generated or analyzed during the current study.
